# Family Health Care Needs in a Pediatric Population 6 Months After Moderate and Severe Physical Trauma

**DOI:** 10.3390/jcm13216490

**Published:** 2024-10-29

**Authors:** Torgeir Hellstrøm, José Luis Castillo Laderas, Håkon Øgreid Moksnes, Audny Anke, Christoph Schäfer, Helene Lundgaard Soberg, Nina Rohrer-Baumgartner, Ingvil Laberg Holthe, Nada Andelic, Mari Storli Rasmussen

**Affiliations:** 1 Department of Physical Medicine and Rehabilitation, Oslo University Hospital, 0424 Oslo, Norway; hakmok@ous-hf.no (H.Ø.M.); christoph.schafer@unn.no (C.S.); helus@oslomet.no (H.L.S.); nadand@ous-hf.no (N.A.); masras@ous-hf.no (M.S.R.); 2 Instituto Universitario de Integración en la Comunidad (INICO), Facultad de Psicología, Universidad de Salamanca, 37005 Salamanca, Spain; joseluisecastilloaderas@usal.es; 3 Institute of Health and Society, Research Centre for Habilitation and Rehabilitation Models & Services (CHARM), Faculty of Medicine, University of Oslo, 0424 Oslo, Norway; audny.anke@uit.no; 4 Department of Clinical Medicine, Faculty of Health Sciences, UiT the Arctic University of Norway, 9019 Tromsø, Norway; 5 Department of Rehabilitation, University Hospital of North Norway, 9019 Tromsø, Norway; 6 Faculty of Health Sciences, Oslo Metropolitan University, 0176 Oslo, Norway; 7 Department of Research and Innovation, Sunnaas Rehabilitation Hospital, 1453 Bjørnemyr, Norway; uxronb@sunnaas.no (N.R.-B.); ingvil.laberg.holthe@sunnaas.no (I.L.H.)

**Keywords:** family needs, pediatric multitrauma, prospective cohort study

## Abstract

**Background**: Traumatic injury is a leading cause of death and disability in children and young adults. There is a lack of evidence-based literature and guidelines on supporting families after severe child injury. This study aimed to assess the family needs and factors associated with those needs. **Methods**: A prospective multicenter follow-up study conducted at two Norwegian trauma centers involving children (aged 0–18 years) who sustained a moderate or severe traumatic injury with a New Injury Severity Score > 9. Sociodemographic and injury variables were recorded at baseline. The Family Needs Questionnaire—Paediatric Version, ranging from one (not at all met) to five (completely met) was completed by parents to assess the family needs at the 6-month follow-up. Bivariate logistic regression analyses were conducted to identify the factors associated with the family needs at 6 months post-injury. **Results:** Of the 63 children included, 38 (68% boys) with a mean age of 9.9 years (SD = 5.8) were available for follow-up. At 6 months, 82% reported needs for health information with a mean score of 3.8 (SD = 1.0), and involvement with care with a mean of 3.7 (SD = 1.2). Additionally, 71% reported emotional support needs (mean score 2.6, SD = 1.3). A higher number of injuries and a lower age of the child were significantly associated with increased odds of having more family needs. **Conclusions**: After moderate to severe pediatric traumatic injury, families report a need for health information, involvement in care, and emotional support. Paying attention to the number of injuries and the child’s age could help to identify families in need of information and support.

## 1. Introduction

Traumatic injury is the leading cause of death and disability in children and young adults [1]. The consequences of injury impact various aspects of quality of life, including physical, emotional, and psychosocial health for the child and their family [2], resulting in different needs.

A synthesis of the international injury literature on the experience of surviving life-threatening injury revealed that individuals of all ages heavily rely on family support throughout the injury trajectory [3]. Caregivers play a crucial role in supporting children following pediatric physical injury, and younger children receive more caregiver involvement in their recovery compared with adolescents [4]. Inadequate parental support negatively affects the physical and psychological adjustment of critically injured children [5,6], and can threaten the well-being of the entire family [7]. Several factors influence how parents respond to their child’s injury, including the severity of the injury, their involvement in the incident, the parent’s mental health and coping strategies, and the normal functioning of the family [8].

A qualitative study of parents’ needs when caring for critically injured children during hospital admission found that the emotional needs of parents, children, and their families were not adequately addressed [9]. A lack of discharge planning, continuity of care, and hospital follow-up were identified. Additionally, the needs of children with traumatic injuries and their families are often unmet, particularly in terms of information, emotional support, and support during care transitions [10]. In another qualitative study on families’ needs following a child’s traumatic injury, the results showed needs for education and training to understand and manage the injury, effective communication, access to sufficient services, support in coordinating care, and positive partnerships with professionals [11]. The families often experience unmet needs for information throughout the continuum of care, access to health services, and inadequate service provision, especially for psychological support [11].

Currently, there is a lack of evidence-based literature and clinical guidelines on supporting parents following critical child injury. Families of children with consequences after traumatic injury may have different needs. To address such specific needs and assess the extent to which they are met, the Family Needs Questionnaire—Pediatric Version (FNQ-P) was developed for the caregivers of children with different types of acquired brain injuries. In this study, we utilized an adapted version of the FNQ-P [12] to assess the needs 6 months after moderate and severe traumatic injuries. The aim of this study was to explore the association between various factors, such as age, length of stay in acute care, number of injuries, severity of injury, and unmet needs 6 months after a traumatic injury.

## 2. Materials and Methods

### 2.1. Design and Setting

This prospective multicenter follow-up study is part of a larger trauma project of all ages in South-East and North Norway [13]. It focuses on children who sustained moderate to severe traumatic injuries and were admitted to Oslo University Hospital (OUH) or the University Hospital of North Norway (UNN). The study was approved by the Norwegian Regional Committee for Medical and Health Research Ethics (approval no. 31676) and the Institutional Data Protection Officers at OUH and UNN (approval numbers 19/26515 and 02423). Children aged 0 to 18 years were included in the study. Informed consent was obtained from parents/legal guardians for children under 16 years, while children aged 16–18 years provided their own informed consent in addition to consent from their parents/legal guardians. At 6 months post-injury, a telephone follow-up was conducted, including the FNQ-P. If preferred, questionnaires were also sent by mail. The Family Needs Questionnaire—P [12] was completed by the parents/legal guardians of the child.

### 2.2. Inclusion and Exclusion Criteria

The inclusion criteria were children who were admitted to OUH or UNN, or transferred from a local hospital within 72 h, with a hospital stay of at least 2 days, and who had sustained a moderate to severe traumatic injury as classified by a New Injury Severity Score (NISS) of >9 [14]. According to the NICE guidelines, patients with an Injury Severity Scale (ISS) score above 9 (in a trauma center) should be assessed for rehabilitation needs [15]. However, in this study, we used NISS scores as their calculation methodology enables a more adequate rating of the severity of a patient presenting multiple injuries and identifies a higher number of major traumas [16]. Patients who were not Norwegian residents, who did not speak Norwegian or English fluently, or who died before discharge were excluded. Children admitted to the regional trauma centers were assessed by medical doctors in the project and the parents of eligible children were invited to participate. The recruitment period at OUH was from 1 January 2020 to 31 December 2020, and at UNN from 1 February 2020 to 31 January 2021. A study protocol has been previously published [13].

### 2.3. Outcome Measures

The outcome measure utilized in this study was the Family Needs Questionnaire—Pediatric Version (FNQ-P) [12]. It is a 40-item scale that assesses the extent to which healthcare family needs are met within six domains: Health Information (10 items); Emotional Support (6 items); Instrumental Support (4 items); Professional Support (6 items); Community Support (6 items); and Involvement with Care (8 items). It also provides a total score of the family needs (40 items). The questionnaire is suitable for both acute and chronic rehabilitation and is based on self-reported information from the child’s parents. Although it was originally developed for pediatric acquired brain injury (ABI), it was used in this study for children with various traumatic injuries. Despite being developed for ABI, most questions are not ABI-specific and thus equally suited for estimating needs after general injury [12]. In agreement with the instrument developers, we replaced “brain injury” with “multitrauma” (License Holland Bloorview Kids Rehabilitation Hospital, 1 April 2020). The FNQ-P was answered on a Likert-type scale ranging from 1 (not at all met) to 5 (completely met) with lower scores indicating more unmet needs. Additionally, respondents had the option to answer “not needed” if an item was not perceived as a current need at the time of completion [12]. International validation of the FNQ-P has shown good test–retest reliability [17], and it has been translated and adapted into Norwegian [18]. The procedure for managing missing data followed a similar approach as used by others [19,20]. Missing data for the FNQ-P domains were imputed with the mean value of that domain when the missing data accounted for less than 25% of the items. The total FNQ-P index was not considered valid if more than one domain was missing.

### 2.4. Patient Characteristics

Patients’ sociodemographic and clinical characteristics were recorded at baseline including age, sex, and geographical home location classified by the Norwegian Centrality Index Score (NCI). The NCI is a coding system that classifies municipal geographical locations in relation to urban areas and their size, ranging from 1 (most central) to 6 (least central) [21]. Additionally, pre-injury comorbidity was categorized using the American Society of Anesthesiologists (ASA)’s Physical Status Classification System [22], with ASA 1 indicating good health and ASA 2–4 indicating the presence of a systemic disease as a measure of pre-injury comorbidities.

### 2.5. Injury and Clinical Characteristics

The cause of injury (fall, transportation, and others) and hospital admission place (local hospital vs. directly from the injury site) were recorded from the medical journals. The number of injuries and injury severity, classified by the ISS and the NISS, were collected from the hospitals’ trauma registries. The ISS and the NISS are anatomical scoring systems that give an overall score for patients with multiple traumas. The NISS score is the sum of squares of the three most severe injuries, independent of the body region. Both the ISS and the NISS range from 0–75 [14]. Data on the injury severity (NISS) and the number of injuries were extracted from the trauma registries of the two hospitals. In this study, we used the NISS scores dichotomized into moderate injury (NISS 10–15) and severe injury (NISS > 15). We also recorded Abbreviated Injury Scores (AISs) for the different body regions, dichotomized into AIS (body region) < 3 and AIS (body region) ≥ 3, where an AIS ≥ 3 is considered a severe injury [14]. Furthermore, non-surgical (no/yes) and surgical (no/yes) procedures were recorded, as well as complications (no/yes), length of acute hospital stay (days), and discharge place (home/local hospitals or rehabilitation).

### 2.6. Statistics

Descriptive data are presented as means and standard deviations (SDs) or medians (IQRs). Proportions are reported for the categorical data. The FNQ-P data were analyzed based on two different groups of variables: the number of healthcare family needs (current needs) and the extent to which the current needs were met (level of needs met), both reported by the parents. For the current needs variables, the percentages were calculated for each domain for those who rated an item between 1 and 5 as ‘a need’ (1), as opposed to those categorized as ‘not a need’ (0) when “not needed” was answered. Therefore, a higher percentage indicates that families had a higher number of needs in that domain. The families were later categorized based on whether they had low or high percentages of current needs in each domain. High and low proportions were determined using the median split percentages of each domain in the present study, as the data were not normally distributed. For the level of needs met, the indexes were calculated for each domain as the mean score of items ranging from 1 (not at all met) to 5 (completely met). The summary scores (means and standard deviations) are also presented by the domain. Univariate and multivariable binary logistic regression analyses were performed to examine the associations between sociodemographic and injury variables, and proportions of current needs (zero: low or one: high) at 6 months post-injury. Due to the limited sample size, only a limited number of independent variables were included in the logistic regression analysis based on clinical importance: age (continuous); number of injuries; overall injury severity as defined by the NISS (<15/≥15); and length of hospital stay. Multicollinearity was assessed using Spearman’s correlation matrix. The results are presented as odds ratios (ORs) with 95% confidence intervals. The model fit is reported using the Hosmer and Lemeshow goodness-of-fit test and Nagelkerke R^2^. The significance level was set at 0.05. All analyses were performed using SPSS Version 28 (IBM Corp: Armonk, NY, USA).

## 3. Results

### Participants

As shown in Table 1, 63 children (67% boys) with a mean (SD) age of 10.3 (5.5) years were initially included in the main study. A total of 38 families (60%) completed the FNQ-P at the 6-month follow-up, (68% boys) with a mean (SD) age of 9.9 (5.8) years and were included in the present study. The majority of children lived in less central areas (63% vs. 37%). The main cause of injury was fall (47%), followed by transportation (34%), and most patients had a severe injury as classified by the NISS. The majority (63%) were admitted to the trauma department from the emergency room/local hospital.

In total, 79% of the families reported having needs in one or several domains at the 6-month follow-up (Table 2). The highest proportion of needs was within the FNQ-P health information and involvement with care domains, followed by the emotional support domain. Looking at the scores indicating the extent to which the needs had been met, with higher scores implying a greater extent of the need being met, the most met needs were within the health information domain (mean = 3.8, SD = 1.0) for items such as being informed about all the changes in my child’s health status in a timely manner, and having information on how the injury will impact my child’s abilities in the future and into adulthood, including information on prognosis and having information from professionals explained in terms and language I can understand. This was followed by involvement with care (mean = 3.7, SD = 0.9) for items such as being involved in planning my child’s transitions, receiving regular communication about my child’s care plan and progress, and being able to review my child’s medical record and ask questions about my child’s diagnosis, physical issues, or thinking challenges. On the contrary, the least met needs were within the emotional support domain (mean = 2.6, SD = 1.3), followed by instrumental support (mean = 2.9, SD = 1.1).

The results of the multiple logistic regression models of the factors associated with the current family needs (low proportion of needs/high proportions of needs as showed in Table 3) are displayed in Table 4 and Table 5 for the FNQ-P total score and Emotional Support needs. See Appendix A (Table A1, Table A2, Table A3, Table A4 and Table A5) for all the FNQ-P domains. The number of injuries was significantly associated with an increased odds of having high levels of total current needs in all the FNQ-P domains except health information needs (Table A1 Appendix A). Age was also a significant predictor, indicating that older age was significantly associated with decreased odds of having more total needs, needs for emotional support (a 1-year increase in age corresponded to a 16% decrease in high levels of needs, OR = 0.84, *p* = 0.042), and professional support (a 1-year increase in age corresponded to a 22% decrease in high levels of needs, OR = 0.78, *p* = 0.027) (Table A4 Appendix A) 6 months after injury. Injury severity, expressed by a NISS ≤ 15 vs. NISS > 15, was not a significant predictor in the multivariate models. Although not statistically significant in the multivariate models, having a more severe injury, expressed as an NISS > 15, increased the odds of high levels of instrumental needs (OR = 2.72, *p* = 0.268) (Table A2 Appendix A) and involvement with care needs (OR = 1.44, *p* = 0.681) (Table A5 Appendix A), and decreased odds of having high levels of health information (OR = 0.786, *p* = 0.765), emotional support (OR = 0.591, *p* = 0.557), and community support needs (OR = 0.697, *p* = 0.672) (Table A3 Appendix A). The length of acute hospital stay was not a significant predictor in the multivariate logistic regression models.

## 4. Discussion

In this study, we examined the family needs, both met and unmet, in a pediatric population with moderate or severe physical trauma, using the FNQ-P. Our findings indicate that a high proportion of the families reported having needs in one or more domains 6 months after the injury. The majority of needs were related to health information, involvement with care, and emotional support. Furthermore, a higher number of injuries was a significant predictor of higher levels of total needs, emotional support needs, professional support needs, and involvement with care needs. Older age was significantly associated with a decreased odds of having high levels of current professional and emotional support needs. The least met needs were within the emotional support domain.

The literature on family needs for children with moderate or severe traumatic injury is scarce, focusing mainly on children with TBI. We found that approximately 80% of the families had needs for health information, followed by involvement with care and emotional support. This is in line with a previous study that identified the need for health information as one of the most important service-related needs following childhood traumatic injury [10]. Although a large proportion of the families in the present study reported needs for health information, these were the needs that were rated as most met. The items within the health information domain that were most often rated as met were: to have information from professionals explained in terms and in a language I can understand; and to be told about all the changes in my child’s health status in a timely manner. This contrasts the scoping review by Jones et al., where health information needs were rated as largely unmet [10]. They also noted that emotional support needs were frequently described but often unrecognized and unmet [10]. Similarly, our results showed that emotional support needs were among the most frequently reported needs at 6 months after injury and that these needs were rated the least met. This suggests that while families’ needs for health information may be adequately addressed, needs for emotional support remain unmet and should be addressed more extensively during the acute and sub-acute recovery phases following child traumatic brain injury. Although not a significant predictor, we cannot dismiss the potential explanation that the children primarily resided in less central areas, as there may be greater access to relevant services in more central areas. Since caregivers’ mental health is strongly associated with children’s emotional and behavioral recovery [23,24], it is crucial to identify the barriers and facilitators to their families’ emotional recovery. This understanding can help to facilitate recovery and improve overall functioning.

The findings of this study are consistent with several studies using the revised adult version of the FNQ in the TBI population. In a Norwegian multicenter study on adults (>15 y) with severe TBI, the most frequently met family needs were on the Health Information subscale, while the most frequently unmet needs were on the Emotional support subscale [20]. Similarly, in a study from the US, needs related to the Health Information subscale were most frequently rated as met, while needs related to the Instrumental Support and Emotional Support subscales were most frequently rated as unmet [19]. This suggests that family needs, when measured with the FNQ, may be similar in TBIs and other traumatic injuries, as well as across the pediatric and adult populations.

The association between needs and the number of injuries could indicate that the extent of the injury increases the family burden. The number of injuries is shown to be associated with lower functional outcomes both at 6 and 12 months, potentially increasing the burden [25,26]. Additionally, parents’ experiences and needs are closely related to their child’s physical and emotional recovery, as well as the support services available to them [27]. Parents report that the impact of their child’s emotional recovery and mental health following the injury are linked to their physical recovery [27]. In this study, we did not find significant association between overall injury severity, as classified by the NISS, and the FNQ-P; however, the odds ratios of instrumental needs (OR = 2.72) and involvement with care (OR = 1.44) were increased in those with more severe injuries. This can suggest that families with a more severely injured child needed more practical support in the rehabilitation process, as well as family-centered services such as involvement in care, transition, and treatment programs in line with other studies [28]. In a study on general traumatic injury in children, the total service cost for caregivers increased with the severity of the injury, as measured by the ISS and age [29]. Although that study was conducted 8–10 years after the accident, so it was cross-sectional and retrospective making it less comparable with our results, the number of injuries is a factor that could contribute to the total burden of injury.

The number of injuries increased the odds for emotional support needs. In a previous qualitative study, caregivers reported different emotional needs while their child was in the hospital and difficulties in adjusting after discharge [30]. After discharge, most caregivers noted that their child experienced symptoms such as anxiety, stress, irritability, depression, and a lack of interest in previously enjoyable activities. Caregivers also mentioned being surprised by their own emotional reactions to their child’s injury, stating that they were unprepared to cope with their emotions. Additionally, caregivers expressed concerns about follow-up medical care, uncertainty about how to care for their child without the assistance of a medical team, and stress about the long-term consequences of the injury. Barriers to emotional recovery, including limited access to medical assistance and challenges in resuming a normal routine with new physical limitations were identified [30]. These findings emphasize the importance of timely education, assessment, and early intervention targeting caregiver and child acute stress and mood-related symptoms in routine patient care.

The number of injuries was also associated with high level of professional support needs. More injuries may lead to greater disability, thereby increasing the need for professionals to compensate for these disabilities. Community support and involvement in care were also linked to the number of injuries. A scoping review by Jones et al. emphasized the need for support with cognitive, emotional, and social problems, which were frequently unrecognized and unmet [10]. This was true for both children with orthopedic injuries and those with traumatic brain injury. Children required assistance with feelings of frustration and depression, stemming from their inability to do what they could before their injury and from being bullied [10].

The literature addresses person-related needs, including physical problems and practical difficulties [10]. Parents require help in balancing their time between caring for their injured child and other work and home responsibilities. Families and friends often provide assistance [9]. Injured children and their families perceive transitions between care settings, such as hospital discharge and return to school, as critical times when their needs are often unmet [9,31]. Specifically, they find information on community and educational services inadequate, exacerbated by a lack of coordination between families, healthcare professionals, and educational services [9,31]. As a result, referrals from specialist trauma centers to primary care, community, social, and educational services are often not made, leading to significant difficulties in accessing the necessary support and services for injured children and their families [31,32,33]. Furthermore, community staff often lack a sufficient understanding of the child’s injuries and their impact, hindering their ability to support the child’s return to everyday activities [31,32,33,34].

We found that age tended to or was significantly associated with total, emotional, and professional support needs. Lower age was associated with a higher odds of unmet emotional and professional support needs. Younger age has been linked to greater caregiver involvement in pediatric traumatic injury recovery [4,30]. Almost half of the patients included in this study had an AIS score ≥ 3 for head injury. Traumatic brain injury during childhood can result in long-term functional, cognitive, behavioral, and psychosocial impairments [35] requiring families to seek assistance with cognitive, emotional, and social challenges. Unfortunately, these problems often go unnoticed and unaddressed [10]. Parents may also experience uncertainty about the child’s future development, leading to higher levels of needs for emotional and professional support [32].

Few studies have explored family needs following child traumatic injury using the FNQ-P, making this study one of the first to assess family needs following moderate to severe traumatic injury in children. This study has some limitations, including a relatively small follow-up rate on the FNQ-P, with only 60% of included families completing the questionnaire at the 6-month follow-up. This resulted in a small sample size, reducing the statistical power of the analyses. Additionally, the small sample size in an otherwise heterogeneous sample can impact the representativeness and generalizability of the findings. The study results should be validated in larger samples and focus on identifying how family needs may change throughout different phases of recovery. Qualitative studies that explore the family’s needs in greater depth are warranted. While the clinical setting considers the injured child and their family as a single unit, it cannot be assumed that families accurately convey the child’s perspectives [36]. Further efforts to gather and report the views of injured children are also necessary.

## 5. Conclusions

This study examined family needs in a pediatric population with moderate and severe physical trauma. The most common needs reported at 6 months after injury were related to health information, involvement with care, and emotional support. Families with younger children and more injuries had higher total needs and greater needs for emotional and professional support. The results provide insight into the different types of needs that families may experience, which health professionals should consider when providing care and developing tailored treatment programs.

## Figures and Tables

**Table 1:** Sociodemographic, clinical, and injury-related variables for the whole sample and for those with complete data on the FNQ-P at the 6-month follow-up.

Variable	All Participants (*n* = 63)	Included in This Study (*n* = 38)
Sociodemographic variables		
Sex (boy), *n* (%)	42 (66.7)	26 (68.4)
Age, mean (SD)	10.3 (5.5)	9.9 (5.8)
Geographical centrality		
Central, *n* (%)	24 (28.1)	14 (36.8)
Less central, *n* (%)	39 (61.9)	24 (63.2)
Pre-injury health status (ASA), *n* (%)	63 (100)	38 (100)
ASA 1 (healthy)	-	-
ASA 2–4 (comorbidity)		
Injury-related variables		
Cause of injury, *n* (%)		
Fall	26 (41.3)	16 (47.4)
Transportation	22 (34.9)	13 (34.2)
Other	15 (23.8)	7 (18.4)
Admission to trauma center, *n* (%)		
Admitted from injury site	31 (49.2)	14 (36.8)
Admitted from emergency room/local hospital	32 (50.8)	24 (63.2)
Number of injuries, mean (SD)	5.7 (3.7)	5.8 (3.7)
Injury Severity Score (ISS), mean (SD)	17.6 (9.6)	17.7 (9.3)
New Injury Severity Score (NISS), *n* (%)		
NISS 10–15	17 (27.0)	10 (26.3)
NISS >15	46 (73.0)	36 (57.1)
Head AIS ≥ 3, *n* (%)	36 (57.1)	17 (44.7)
Neck AIS ≥ 3, *n* (%)	1 (1.6)	1 (2.6)
Abdomen AIS ≥ 3, *n* (%)	15 (23.8)	8 (21.1)
Spine AIS ≥ 3, *n* (%)	6 (9.5)	5 (13.2)
Extremity AIS ≥ 3, *n* (%)	6 (9.5)	3 (7.9)
Non-surgical procedure, *n* (%)	47 (74.6)	26 (68.4)
Surgical procedure (yes), *n* (%)	34 (54.0)	17 (44.7)
* LOS in days, mean (SD)	8.7 (13.8)	6.8 (6.8)
Discharge place, home/local hospital,		
*n* (%)	46 (73.0)	26 (68.4)
Discharge place, rehabilitation, *n* (%)	17 (27.0)	12 (31.6)

* LOS, length of acute hospital stay.

**Table 2:** Percentages and means (SDs) on the FNQ-P at 6 months post-injury and mean (SD) ratings on the FNQ-P (*n* = 38).

Domain	Current Needs (%)	Mean (SD) Ratings on the FNQ-P
Health information	81.6%	3.8 (1.0) (*n* = 31)
Emotional support	71.1%	2.6 (1.3) (*n* = 27)
Instrumental support	57.9%	2.9 (1.1) (*n* = 22)
Community support	65.8%	3.1 (1.2) (*n* = 25)
Professional support	58.4%	3.4 (1.3) (*n* = 26)
Involvement with care	81.6%	3.7 (1.2) (*n* = 31)
Total *	78.9%	3.5 (0.9) (*n* = 30)

* Total percentage was calculated according to the number of participants with all data available.

**Table 3:** *n* (%) of families with low and high proportions of current needs on the FNQ-P at 6 months post-injury.

Domain	Current Needs	*n* (%)
Health information	Low High	18 (47.4) 20 (52.6)
Emotional support	Low High	19 (50.0) 19 (50.0)
Instrumental support	Low High	16 (42.1) 22 (57.9)
Community support	Low High	13 (34.2) 25 (65.8)
Professional support	Low High	17 (45.9) 20 (54.1)
Involvement with care	Low High	19 (50.0) 19 (50.0)
Total	Low High	19 (50.0) 19 (50.0)

**Table 4:** Results from the multivariate logistic regression analyses of the FNQ-P total score on total current needs at the 6-month follow up (*n* = 38).

Variable	OR	95% CI	*p*-Value
Age	0.854	0.725–1.005	0.058
Number of injuries	**1.495**	**1.051–2.126**	**0.025**
NISS (<15 ^a^/≥15)	1.312	0.228–2.126	0.761
Length of stay	1.042	0.884–1.227	0.626
Hosmer and Lemeshow X^2^ 11.794, df 8, *p*-value 0.161; Nagelkerke X^2^ 0.432			

^a^ = reference category; OR > 1 increases the odds of having a high level of current needs; OR < 1 decreases the odds of having a high level of current needs. Statistically significant results are marked in bold.

**Table 5:** Results from the multivariate logistic regression analyses of the FNQ-P score on current emotional support needs at the 6-month follow-up (*n* = 38).

Variable	OR	95% CI	*p*-Value
Age	**0.843**	**0.716–0.994**	**0.042**
Number of injuries	**1.616**	**1.113–2.347**	**0.012**
NISS (<15 ^a^/≥15)	0.591	0.102–3.414	0.557
Length of stay	0.990	0.854–1.147	0.892
Hosmer and Lemeshow X^2^ 5.528, df 8, *p*-value 0.700; Nagelkerke X^2^ 0.432			

^a^ = reference category; OR > 1 increases the odds of having a high level of current needs; OR < 1 decreases the odds of having a high level of current needs. Statistically significant results are marked in bold.

## Data Availability

The datasets generated and/or analyzed in the current study are not publicly available due to the sensitivity of the material.

## Appendix A

**Table A1:** Results from the multivariate logistic regression analyses of the FNQ-P score on current health information needs at the 6-month follow-up (*n* = 38).

Variable	OR	95% CI	*p*-Value
Age	0.972	0.862–1.095	0.639
Number of injuries	1.213	0.944–1.560	0.132
NISS (<15 ^a^/≥15)	0.786	0.162–3.808	0.765
Length of stay	0.956	0.846–1.080	0.466
Hosmer and Lemeshow X^2^ 7.296, df 8, *p*-value 0.505; Nagelkerke X^2^ 0.089			

^a^ = reference category; OR > 1 increases the odds of having a high level of current needs; OR < 1 decreases the odds of having a high level of current needs.

**Table A2:** Results from the multivariate logistic regression analyses of the FNQ score on current instrumental support needs at the 6-month follow-up (*n* = 38).

Variable	OR	95% CI	*p*-Value
Age	0.911	0.777–1.067	0.248
Number of injuries	1.454	0.986–2.142	0.059
NISS (<15 ^a^/≥15)	2.718	0.464–15.932	0.268
Length of stay	1.123	0.901–1.399	0.303
Hosmer and Lemeshow X^2^ 6.210, df 8, *p*-value 0.624; Nagelkerke X^2^ 0.428			

^a^ = reference category; OR > 1 increases the odds of having a high level of current needs; OR < 1 decreases the odds of having a high level of current needs.

**Table A3:** Results from the multivariate logistic regression analyses of the FNQ-P score on current community support needs at the 6-month follow-up (*n* = 38).

Variable	OR	95% CI	*p*-Value
Age	0.938	0.813–1.082	0.377
Number of injuries	**1.445**	**1.008–2.071**	**0.045**
NISS (<15 ^a^/≥15)	0.697	0.132–3.692	0.672
Length of stay	0.960	0.827–1.113	0.586
Hosmer and Lemeshow X^2^ 4.368, df 8, *p*-value 0.737; Nagelkerke X^2^ 0.225			

^a^ = reference category; OR > 1 increases the odds of having a high level of current needs; OR < 1 decreases the odds of having a high level of current needs. Statistically significant results are marked in bold.

**Table A4:** Results from the multivariate logistic regression analyses of the FNQ-P score on current professional support needs at the 6-month follow-up (*n* = 38).

Variable	OR	95% CI	*p*-Value
Age	**0.782**	**0.629–0.973**	**0.027**
Number of injuries	**2.105**	**1.201–3.690**	**0.009**
NISS (<15 ^a^/≥15)	0.786	1.201–3.690	0.786
Length of stay	0.941	0.791–1.119	0.490
Hosmer and Lemeshow X^2^ 9.976, df 8, *p*-value 0.190; Nagelkerke X^2^ 0.546			

^a^ = reference category; OR > 1 increases the odds of having a high level of current needs; OR < 1 decreases the odds of having a high level of current needs. Statistically significant results are marked in bold.

**Table A5:** Results from the multivariate logistic regression analyses of the FNQ-P score on current involvement with care needs at the 6-month follow-up (n = 38).

Variable	OR	95% CI	*p*-Value
Age	0.892	0.768–1.036	0.136
Number of injuries	**1.534**	**1.083–2.173**	**0.016**
NISS (<15 ^a^/≥15)	1.444	0.251–8.314	0.681
Length of stay	0.952	0.827–1.095	0.489
Hosmer and Lemeshow X^2^ 13.512, df 8, *p*-value 0.095; Nagelkerke X^2^ 0.371			

^a^ = reference category; OR > 1 increases the odds of having a high level of current needs; OR < 1 decreases the odds of having a high level of current needs. Statistically significant results are marked in bold.
